# Reliability of genomic prediction for milk fatty acid composition by using a multi-population reference and incorporating GWAS results

**DOI:** 10.1186/s12711-019-0460-z

**Published:** 2019-04-27

**Authors:** Grum Gebreyesus, Henk Bovenhuis, Mogens S. Lund, Nina A. Poulsen, Dongxiao Sun, Bart Buitenhuis

**Affiliations:** 1 Department of Molecular Biology and Genetics, Center for Quantitative Genetics and Genomics, Aarhus University, Blichers Allé 20, P.O. Box 50, 8830 Tjele, Denmark; 2 Animal Breeding and Genomics Centre, Wageningen University, P.O. Box 338, 6700 AH Wageningen, The Netherlands; 3 Department of Food Science, Aarhus University, Blichers Allé 20, P.O. Box 50, 8830 Tjele, Denmark; 4 Laboratory of Animal Genetics, Breeding and Reproduction, Ministry of Agriculture of China, National Engineering Laboratory for Animal Breeding, College of Animal Science and Technology, China Agricultural University, Beijing, 100193 China

## Abstract

**Background:**

Large-scale phenotyping for detailed milk fatty acid (FA) composition is difficult due to expensive and time-consuming analytical techniques. Reliability of genomic prediction is often low for traits that are expensive/difficult to measure and for breeds with a small reference population size. An effective method to increase reference population size could be to combine datasets from different populations. Prediction models might also benefit from incorporation of information on the biological underpinnings of quantitative traits. Genome-wide association studies (GWAS) show that genomic regions on *Bos taurus* chromosomes (BTA) 14, 19 and 26 underlie substantial proportions of the genetic variation in milk FA traits. Genomic prediction models that incorporate such results could enable improved prediction accuracy in spite of limited reference population sizes. In this study, we combine gas chromatography quantified FA samples from the Chinese, Danish and Dutch Holstein populations and implement a genomic feature best linear unbiased prediction (GFBLUP) model that incorporates variants on BTA14, 19 and 26 as genomic features for which random genetic effects are estimated separately. Prediction reliabilities were compared to those estimated with traditional GBLUP models.

**Results:**

Predictions using a multi-population reference and a traditional GBLUP model resulted in average gains in prediction reliability of 10% points in the Dutch, 8% points in the Danish and 1% point in the Chinese populations compared to predictions based on population-specific references. Compared to the traditional GBLUP, implementation of the GFBLUP model with a multi-population reference led to further increases in prediction reliability of up to 38% points in the Dutch, 23% points in the Danish and 13% points in the Chinese populations. Prediction reliabilities from the GFBLUP model were moderate to high across the FA traits analyzed.

**Conclusions:**

Our study shows that it is possible to predict genetic merits for milk FA traits with reasonable accuracy by combining related populations of a breed and using models that incorporate GWAS results. Our findings indicate that international collaborations that facilitate access to multi-population datasets could be highly beneficial to the implementation of genomic selection for detailed milk composition traits.

**Electronic supplementary material:**

The online version of this article (10.1186/s12711-019-0460-z) contains supplementary material, which is available to authorized users.

## Background

Milk contains various fatty acids (FA) that are grouped into different categories depending on the length of the carbon chains, degree of unsaturation and isomerization. Some groups of dietary FA are linked to cardiovascular disease risks, whereas others are suggested to be beneficial to human health [1–3]. Such links have long triggered interests for the modification of the FA profile of bovine milk. Several studies have reported substantial genetic variation in bovine milk FA traits [4, 5], which may provide the opportunity to modify milk FA composition through selective breeding. Genomic selection has become the main strategy in livestock selective breeding by allowing the selection of candidate bulls at younger ages [6]. However, prediction accuracy for traits that are difficult and expensive to measure is still limited because of the small size of reference populations. To date, genomic prediction accuracies have not been reported for milk FA composition traits, in spite of the growing interest to include these in the breeding goals of dairy cattle [7]. This is mainly due to the difficulty of large-scale recording of milk FA traits. Gas chromatography (GC), the current method of choice for quantifying milk FA traits with high accuracy, requires expensive equipment and time-consuming techniques that challenge large-scale phenotyping.

A strategy that is increasingly receiving attention in genomic prediction for numerically small breeds or difficult-to-measure traits is to combine datasets from different breeds/populations [8, 9]. The benefits of combining data for genetic analyses depend highly on the genetic distance between the populations that are used in different studies, and on marker density [8]. In this study, we combined samples for 16 FA traits that were measured by GC in the Chinese, Danish and Dutch Holstein populations that were genotyped using, or imputed to, high-density (HD) single nucleotide polymorphism (SNP) arrays for genomic prediction. Given the common use of outstanding North American bulls in the Chinese, Danish and Dutch Holstein breeding populations, high genetic similarities are expected between these populations. Previous studies showed high consistency in linkage disequilibrium (LD) patterns between the Danish and Chinese Holstein [10, 11] and between the Dutch, Danish and Chinese Holstein populations (Gebreyesus et al. personal communication).

Although genomic prediction allows the use of all genome-wide markers without the need to map quantitative trait loci (QTL), incorporation of biological information might improve furthermore the accuracy of genomic prediction for small-scale recorded traits. Methods have been suggested to weigh variants according to prior knowledge of their effect on the traits [12, 13], and gains in prediction accuracies have been reported [14, 15]. For several years, genome-wide association studies (GWAS) have been used as a powerful tool to investigate the genetic background of quantitative traits and diseases. Incorporation of GWAS results in genomic prediction models can improve genomic prediction accuracy [16], especially when prediction accuracy is limited by reference size. However, a major hurdle in GWAS is that their power of detection depends on the size of the samples available for the analyses. Moreover, GWAS often result in the detection of large genomic regions, especially in cattle breeds, because of long range LD [17]. An option to deal with the limitation in sample size could be to combine several smaller datasets that are available across populations for joint GWA. Additional evidence from gene ontology and pathways analyses might also help refine GWAS detections.

GWAS on milk FA traits have frequently reported significant associations in large regions on *Bos taurus* chromosomes (BTA) 14, 19 and 26 [18–21]. Further studies on the characterization of these regions have also suggested that they have large effects on most milk FA traits [22–27]. In addition, several other regions that explain relatively smaller proportions of the genetic variance in multiple FA traits have been reported across the bovine genome [19, 20]. Information on such major regions that underlie their genetic variation might improve accuracy of genomic prediction for the small-scale recorded milk FA traits.

Traditionally, the genomic best linear unbiased prediction (GBLUP) model [28] is based on the implicit assumption that many QTL, each one explaining a small fraction of the genetic variance, underlie quantitative traits. In the implementation of GBLUP, genetic effects are estimated based on the realized relationship matrix that is computed from genome-wide markers [28]. Often, the contribution of genetic markers to the genomic relationship is not weighted according to the explained proportion of the genetic variance. Such an approach, in which each marker is assumed to contribute equally to the relationship matrix although the associations between genetic variants and traits differ, can cause a “dilution” of the effects of major regions. In this context, Sørensen et al. [29] suggested an extension of the GBLUP model to allow the incorporation of available information about the biological mechanisms that underlie quantitative traits. To implement such an extension, Sørensen et al. [29] suggested a genomic feature BLUP approach (GFBLUP), in which variants are categorized according to biological information, such as chromosomes, genes or biological pathways, so that groups of SNPs are differentiated depending on the variance they explain and the size of their effects for genomic prediction.

In this study, we implemented a GFBLUP approach in which regions that had been detected through GWAS on BTA14, 19, 26 were fitted as genomic features of interest to predict genomic breeding values (GBV) for the FA traits analyzed. Then, we compared these estimated prediction reliabilities to those estimated with the traditional GBLUP model. The objectives of this study were to (1) estimate and compare genomic prediction reliabilities for 16 milk FA traits in three Holstein populations; and (2) investigate gains in genomic prediction reliability from combining multi-population reference sets and incorporating biological information based on GWAS results.

## Methods

### Animals and phenotypes

Milk samples were obtained from 784 Chinese (from 18 herds in China), 675 Danish (from 22 herds in Denmark) and 1566 Dutch Holstein (from 398 herds in the Netherlands) cows. Stages of lactation of the sampled cows ranged from 3 to 700 days in milk (DIM) in the Chinese population, 9 to 481 DIM in the Danish population and 60 to 278 DIM in the Dutch Holstein population. To standardize the samples from these three countries, only cows at 60 DIM or more were considered for genomic prediction, which resulted in 700 Chinese, 614 Danish and 1566 Dutch samples available for the analysis. The reason for restricting samples at lactation stages of 60 days or more is that the genetic determinism of milk fat composition traits may differ in the earlier stages of lactation. Indeed, there is evidence that the effects of genes in early and later lactation differ [30] and excluding early lactation records should help eliminate this heterogeneity issue. All sampled cows in the Dutch Holstein population were at first parity, whereas cows in the Chinese and Danish populations were at parities 2 to 6 and parities 1 and 2, respectively.

Gas chromatography was used to quantify 13 FA traits that were measured as weight proportion of total fat (% wt/wt) (see Table 1) as described by Li et al. [21] for the Chinese samples, Poulsen et al. [31] for the Danish samples and Stoop et al. [4] for the Dutch samples. Furthermore, desaturation indexes were calculated based on the FA measurements as: C14 index = C14:1/(C14:1 + C14:0) × 100; C16 index = C16:1/(C16:1 + C16:0) × 100 and C18 index = C18:1c9/(C18:1c9 + C18:0) × 100.

**Table 1:** Phenotypic means and coefficients of variation (%) for FA traits across populations and combined datasets

FA	CN			DK			NL			Combined		
	Mean	CV	*h* ^2^	Mean	CV	*h* ^2^	Mean	CV	*h* ^2^	Mean	CV	*h* ^2^
Saturated FA^a^												
C8:0	0.58	37.9	0.06	1.47	15.0	0.33	1.31	13.0	0.48	1.18	32.2	0.27
C10:0	2.22	18.0	0.16	3.22	17.4	0.36	2.87	15.7	0.51	2.80	20.7	0.39
C12:0	2.94	16.7	0.21	3.69	18.4	0.30	3.79	19.0	0.40	3.58	21.2	0.33
C14:0	10.10	11.3	0.22	11.60	11.7	0.14	11.10	9.5	0.39	11.00	11.5	0.25
C15:0	0.99	13.1	0.10	1.11	17.1	0.27	1.11	17.1	0.29	1.09	16.5	0.23
C16:0	32.90	5.6	0.27	30.10	11.6	0.12	29.10	12.0	0.48	30.20	11.7	0.34
C18:0	12.00	14.1	0.25	9.84	19.4	0.23	9.84	17.7	0.37	10.30	19.3	0.25
Unsaturated FA^a^												
C14:1	0.86	24.4	0.35	1.01	27.7	0.49	1.38	19.6	0.55	1.19	29.4	0.47
C16:1	1.64	20.1	0.26	1.58	26.6	0.42	1.39	20.9	0.65	1.49	23.5	0.46
C18:1c9	28.30	8.6	0.24	19.60	14.5	0.07	20.20	13.8	0.41	21.90	20.0	0.27
C18:2n6	3.99	11.5	0.26	1.74	15.5	0.17	1.11	22.5	0.27	1.89	63.0	0.18
C18:3n3	0.42	14.3	0.05	0.50	18.0	0.05	0.50	32.0	0.27	0.48	27.1	0.19
CLA	0.41	22.0	0.15	0.57	26.3	0.11	0.56	46.4	0.32	0.53	43.4	0.21
Desaturation indexes^b^												
C14 index	7.84	20.8	0.36	7.98	23.7	0.59	11.0	16.6	0.62	9.71	24.4	0.53
C16 index	4.74	19.6	0.24	4.97	22.3	0.37	4.6	19.8	0.55	4.70	20.6	0.38
C18 index	70.20	4.7	0.21	66.60	5.9	0.26	67.3	5.8	0.49	67.80	5.87	0.31

All parameter estimates for C18:2n6, C18:3n3 and CLA are computed on raw data before log-transformation

^a^Expressed in % wt/wt of total fat

^b^Desaturation indexes calculated as unsaturated/(unsaturated + saturated) × 100

### Genotypes and imputation

Real or imputed high-density (HD) genotypes were available for all sampled cows. All the cows in the Chinese dataset were genotyped using the BovineSNP50 Beadchip (50 K, Illumina). A population of 96 Chinese Holstein bulls, which were genotyped using the BovineHD Beadchip (777 K), was used as reference to impute the 50 K genotypes to HD. Of the 675 Danish cows, 278 were genotyped using the BovineSNP50 Beadchip and 397 were genotyped using the BovineHD Beadchip, which were then used as reference to impute the 50 K genotypes of the first 278 Danish cows to HD as described in Gebreyesus et al. [32]. A custom 50 K SNP Beadchip was used to genotype cows in the Dutch dataset. A reference population consisting of 1333 Dutch Holstein cows and 55 bulls that were genotyped with the BovineHD Beadchip (777 K) was used to subsequently impute the 50 K genotypes of the Dutch samples to HD as described in Duchemin et al. [33].

Quality controls on SNPs were undertaken within each population. Accordingly, SNPs with minor allele frequencies (MAF) lower than 0.05 or with a count of one of the genotypes less than 10 in each population were excluded from both the population-specific as well as combined-population predictions. Finally, 464,130 common SNPs were available for all the populations and scenarios.

### Models

Traditional and “genomic features” GBLUP models were implemented to estimate genomic breeding values (GBV).

#### Traditional GBLUP

GBLUP models were implemented using DMU [34] in two scenarios: (1) population-specific reference sets within the Chinese, Danish and Dutch samples, and (2) a combined reference set of the three populations. The general model used for the traditional GBLUP, both population-specific as well as combined-population reference sets, was:

1 $$y_{ijkl} = \mu + parity_{i} + herd_{j} + b_{1} DIM_{k} + b_{2} \times exp^{{ - 0.05 \times DIM_{k} }} + g_{l} + e_{ijkl} ,$$

where $y_{ijkl}$ is the phenotype of cow $l$ in parity $i$, and herd $j$, $\mu$ is the fixed mean effect; $b_{1}$ is the regression coefficient for $k$th day of lactation $\left( {DIM_{k} } \right)$, $b_{2}$ is the regression coefficient for the Wilmink adjustment ($exp^{{ - 0.05 \times DIM_{k} }}$) of DIM [35], $e_{ijkl}$ is a random residual effect assumed to be normally distributed with ${\mathbf{e}} \sim N\left( {0, {\mathbf{I}} _{e}^{2} } \right)$, where ${\mathbf{I}}$ is an identity matrix. Season of sampling was not modeled in the analyses because all the cows were sampled during the summer. $g_{l}$ is the random additive genetic effect of cow $l$ following a normal distribution $N\left( {0, {\mathbf{G}}\sigma_{a}^{2} } \right),$ where ${\mathbf{G}}$ is the genomic relationship matrix between individuals and $\sigma_{a}^{2}$ is the genetic variance. The genomic relationship matrix used in the GBLUP models was calculated as described in the first method presented by VanRaden [28].

Homogeneity of residuals was assessed by plotting the residuals against predicted phenotypes from Model (1) based on the combined-population dataset with all available individuals. For some of the FA traits, especially for C18:2n6, C18:3n3 and CLA, residuals tend to increase with the mean, which indicates heterogeneity in the residual variance, thus records for these traits were log-transformed for the genomic prediction scenarios.

#### GFBLUP

A GFBLUP model was implemented using a combined-population reference set to estimate GBV for population-specific validation sets. In the traditional GBLUP model, a single random genetic effect based on the genomic relationship matrix that was constructed using all the SNPs was considered. In contrast, four random genetic effects were considered in the GFBLUP approach according to the following model:

2 $$\begin{aligned} y_{ijkl} & = \mu + parity_{i} + herd_{j} + b_{1} DIM_{k} \\ & \quad + b_{2} \times \exp^{{ - 0.05 \times DIM_{k} }} + g_{{14_{l} }} + g_{{19_{l} }} + g_{{26_{l} }} + g_{{R_{l} }} + e_{ijkl} , \\ \end{aligned}$$

where $g_{{14_{l} }}$ is the random additive genetic effect of cow $l$ based on the genomic relationship matrix (${\mathbf{G}}_{14}$) constructed using only the SNPs on BTA14 and assuming a normal distribution with $N\left( {0, {\mathbf{G}}_{14} \sigma_{14}^{2} } \right)$, where $\sigma_{14}^{2}$ is the genetic variation explained by the SNPs on BTA14. Similarly, $g_{{19_{l} }}$ and $g_{{26_{l} }}$ are the random additive genetic effects of cow $l$ based on the relationship matrices computed using the SNPs on BTA19 and 26, respectively, and assumed to follow normal distributions as for $g_{{14_{l} }}$. Finally, $g_{{R_{l} }}$ is the random additive genetic effect of cow $l$ based on the genomic relationship matrix that was constructed by using all remaining SNPs except those on BTA14, 19 and 26, following a normal distribution $N\left( {0, {\mathbf{G}}_{{\mathbf{R}}} \sigma_{R}^{2} } \right)$. The SNPs used to calculate the relationship matrices include 13,033 SNPs on BTA14, 12,603 SNPs on BTA19 and 9703 SNPs on BTA26. The different genomic relationship matrices for the GFBLUP model were computed following the first method of VanRaden [28]. The other components in Model (2) are as defined in Model (1).

The total genomic value was calculated as: $g_{l} = g_{{14_{l} }} + g_{{19_{l} }} + g_{{26_{l} }} + g_{{R_{l} }}$.

The proportion of the genomic variance explained by each genetic component of the GFBLUP model was computed as:

3 $$\% var_{{feature_{i} }} = \frac{{\sigma_{{feature_{i} }}^{2} }}{{\sigma_{total}^{2} }} \times 100,$$

where $\sigma_{{feature_{i} }}^{2}$ is $\sigma_{14}^{2} ,$ or $\sigma_{19,}^{2}$ or $\sigma_{26}^{2}$, and $\sigma_{total}^{2}$ is the total additive genetic variance computed as:

4 $$\sigma_{total}^{2} = \sigma_{14}^{2} + \sigma_{19}^{2} + \sigma_{26}^{2} + \sigma_{R}^{2} .$$

The proportions of genomic variance were estimated based on the combined-population dataset including all available individuals, i.e., both the training and validation sets.

### Binwise linkage disequilibrium on BTA14, 19 and 26

To study the similarity of the LD structures of the bovine chromosomes taken as features in the three populations, pairwise LD was calculated between the SNPs within a 1-Mbp window on BTA14, 19 and 26, with the r^2^ as a measure in the Plink program [36].

### Training and validation populations

For all the scenarios, a resampling strategy was applied to create five validation sets of 100 cows for each population. The general principle was to avoid sibling and dam-progeny relationships between validation sets and with the reference population for each replicate. Table 2 shows the size of the reference populations used in the within- and the combined-population genomic prediction for each trait and population. For the Danish population, a subset of cows that had no siblings and whose dams are not within the dataset were first selected (n = 197). For each analysis of resampled datasets, 100 of these cows were randomly sampled for the validation set and the remaining cows were included back to the reference population. In the Dutch and Chinese populations, all the sampled cows had at least one half-sib in the dataset. In the Dutch dataset, all cows were in first parity and belonged to one of three sire-groups, whereas in the Chinese dataset, the majority of the cows were from five different sires. Hence, a sire-group (group of cows with common sire) was randomly selected for each validation set. Since each sire-group included more than 100 cows in both the Chinese and Dutch datasets, another random sampling of 100 cows was undertaken within the selected sire-groups and the remaining cows from the group were excluded from the reference population. The main reason for restricting the validation to 100 cows was to have comparable reference and validation group sizes in the three populations.

**Table 2:** Number of cows in the reference sets for each FA trait in the Chinese (CN), Danish (DK), Dutch (NL) and the combined-population genomic prediction

Trait	CN		DK		NL	
	Single	Combined	Single	Combined	Single	Combined
C8:0	584	2764	518	2771	892	2188
C10:0	585	2767	520	2775	892	2192
C12:0	585	2765	519	2774	892	2190
C14:0	586	2766	519	2774	892	2191
C15:0	583	2751	516	2760	887	2181
C16:0	583	2762	518	2769	892	2186
C18:0	587	2762	518	2771	889	2178
C14:1	584	2761	516	2769	890	2187
C16:1	583	2755	519	2763	887	2185
C18:1c9	585	2765	518	2773	892	2190
C18:2n6	585	2760	518	2768	889	2188
C18:3n3	583	2750	518	2759	885	2180
CLA	580	2750	518	2758	886	2178
C14 index	583	2758	515	2767	890	2184
C16i ndex	580	2750	517	2757	887	2177
C18 index	585	2758	516	2767	889	2185

### Prediction reliability

For all models, prediction reliability for cows was computed as the squared correlation between estimated GBV and the phenotype corrected for fixed effects and scaled by dividing with the estimated heritabilities. Corrected phenotypes were computed based on single-population traditional GBLUP as in Model (1) and used for all scenarios. The heritability estimates used to scale the reliabilities were from the models in the respective scenario using all available individuals. Accordingly, heritability in the traditional GBLUP approaches was computed as:

5 $$h^{2} = \frac{{\sigma_{a}^{2} }}{{\sigma_{a}^{2} + \sigma_{e}^{2} }},$$

and the heritability for the GFBLUP model was computed as:

6 $$h^{2} = \frac{{\sigma_{total}^{2} }}{{\sigma_{total}^{2} + \sigma_{e}^{2} }},$$

where $\sigma_{total}^{2}$ is the summation of the variance explained by each genomic feature as given in Eq. (4).

For population-specific traditional GBLUP models, all available data from the respective populations were used to estimate heritabilities. For the combined-population analyses (both combined GBLUP and GFBLUP models), the combined-population data including all available individuals was used to estimate heritabilities.

Prediction accuracies reported for each scenario are average values of the five replicates (folds).

## Results

### Heritability estimates for milk fatty acid composition traits

Phenotypic means, coefficients of variation (%) and heritability estimates for the FA traits in the different populations and the combined dataset are in Table 1. Overall, phenotypic means were comparable between the Danish and Dutch datasets, whereas those for the Chinese dataset showed larger differences for some of the FA traits. Such differences between the Chinese data on the one hand and the Danish and Dutch on the other hand were observed, in particular, for C8:0, C18:2n6 and C18:1c9. Larger differences were also observed in the estimates of coefficients of variation between the populations for some of the studied traits. In the combined dataset, coefficients of variation ranged from 5.87% (C18 index) to 63.0% (C18:2n6). Similarly, some differences in the estimated heritabilities were observed, with estimates being generally higher for the Dutch dataset followed by those for the Danish dataset.

### Consistency in LD on BTA14, 19 and 26 between populations

**Fig. 1:**
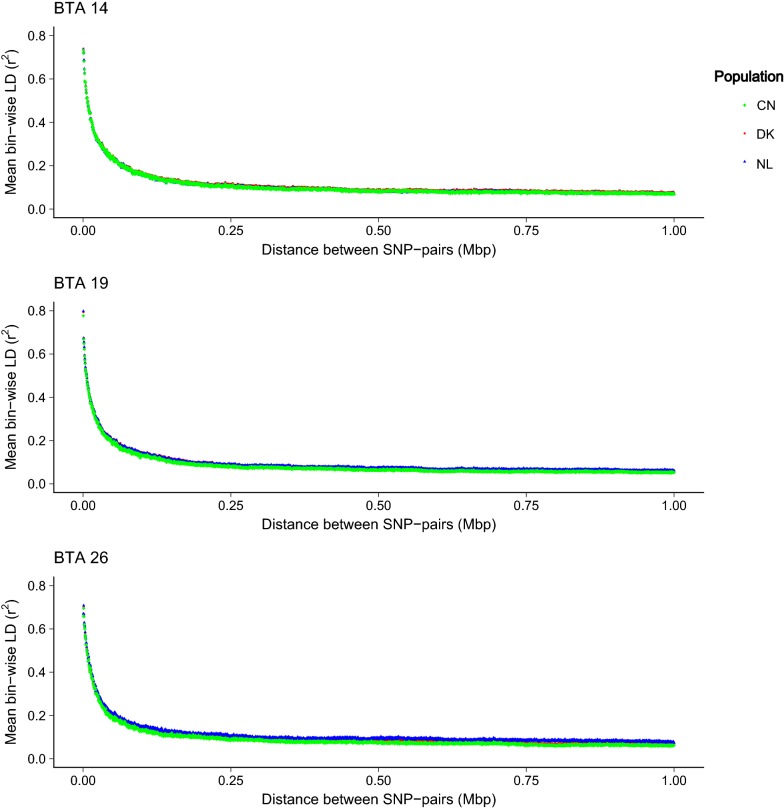
Mean binwise linkage disequilibrium (LD) for the Dutch (blue points), Danish (red points) and Chinese (green points) Holstein Friesian genotypes on BTA14, 19 and 26. The y-axis is the mean binwise LD and the x-axis is the physical distance between pairwise SNPs in mega base pairs (Mbp)

The LD analysis on BTA14, 19 and 26 indicates similar binwise pairwise LD (r^2^) in the three populations within the regions considered for the genomic feature prediction model (Fig. 1). Furthermore, the minimum and maximum average pairwise LD values for SNPs within bins of 1-Mbp sizes were similar between the populations on the three bovine chromosomes.

### Prediction reliability estimated with traditional GBLUP models

Table 3 presents prediction reliabilities for the FA traits studied in the three populations using the GBLUP model based on population-specific reference sets. Prediction reliabilities calculated by using reference populations separately were, in general, low across the FA traits and populations. Prediction reliabilities were especially low for the Chinese dataset followed by those for the Danish population. In the Chinese single-population genomic prediction, reliabilities were highest for C16:0 (0.19) and C18:0 (0.19), followed by C18 Index (0.15). Prediction reliabilities were very low for the de novo synthesized FA in general, and particularly for C10:0, C12:0 and C14:0 in the Chinese validation. Similarly, genomic prediction using the Danish reference population separately resulted in low prediction reliabilities across traits. Prediction reliabilities were highest for CLA (0.14) followed by C14:0 (0.11) and lowest for C16:0 and C18:1c9. Moderate to low prediction reliabilities were computed in the Dutch single-population genomic prediction. Using the Dutch separate reference population, genomic prediction reliability was highest for C14 index (0.43) followed by C14:1 (0.39).

**Table 3:** Genomic prediction reliabilities obtained with traditional GBLUP based on within- and combined-population references

Trait	CN		DK		NL	
	Single	Combined	Single	Combined	Single	Combined
C8:0	0.06	0.05	0.06	0.26	0.11	0.18
C10:0	0.003	0.07	0.06	0.29	0.17	0.28
C12:0	0.001	0.04	0.04	0.18	0.25	0.31
C14:0	0.0004	0.001	0.11	0.11	0.25	0.36
C15:0	0.03	0.03	0.04	0.19	0.03	0.06
C16:0	0.19	0.18	0.001	0.07	0.13	0.19
C18:0	0.19	0.12	0.06	0.07	0.05	0.06
C14:1	0.11	0.15	0.06	0.16	0.39	0.49
C16:1	0.07	0.12	0.09	0.14	0.22	0.39
C18:1c9	0.07	0.15	0.005	0.09	0.13	0.26
C18:2n6	0.06	0.10	0.01	0.03	0.10	0.22
C18:3n3	0.08	0.04	0.10	0.01	0.06	0.21
CLA	0.12	0.10	0.14	0.16	0.16	0.25
C14 index	0.10	0.16	0.05	0.18	0.43	0.56
C16 index	0.10	0.13	0.09	0.14	0.12	0.30
C18 index	0.15	0.05	0.02	0.15	0.12	0.19

Reliabilities of genomic predictions based on the combined-population reference were higher across traits and populations compared to those based on separate reference populations (Table 3). Genomic prediction reliability based on the multi-population reference was on average 10% points higher for the Dutch validation population compared to single-population genomic prediction. Increases in prediction reliability were largest for C16 index (∆ = 0.18), C16:1 (∆ = 0.17) and C18:3n3 (∆ = 0.15), and smallest for C18:0 (∆ = 0.01). In the Danish validation, prediction reliability increased by 8% points on average after adding the Dutch and Chinese reference populations. Increases in prediction reliability were largest for C10:0 (∆ = 0.23), C8:0 (∆ = 0.20) and C15:0 (∆ = 0.15). Sizable improvements in prediction reliability were also observed for C14 and C18 indexes. However, no improvement was obtained for C14:0 and prediction reliability even declined for C18:3n3 with the multi-population prediction compared to the Danish within-population analysis. With an average increase in prediction reliability of 1% point, little or no gain was observed from the combined-population analysis for most FA traits in the Chinese validation. In the Chinese validation, the greatest benefits of adding the Danish and Dutch reference populations were observed for C18:1c9 (∆ = 0.08), followed by C10:0 (∆ = 0.07) and C14 index (∆ = 0.06). However, for some traits, prediction reliabilities were less good with the combined reference than with the single-population reference (Table 3).

### Prediction reliability estimated with the GFBLUP model

**Fig. 2:**
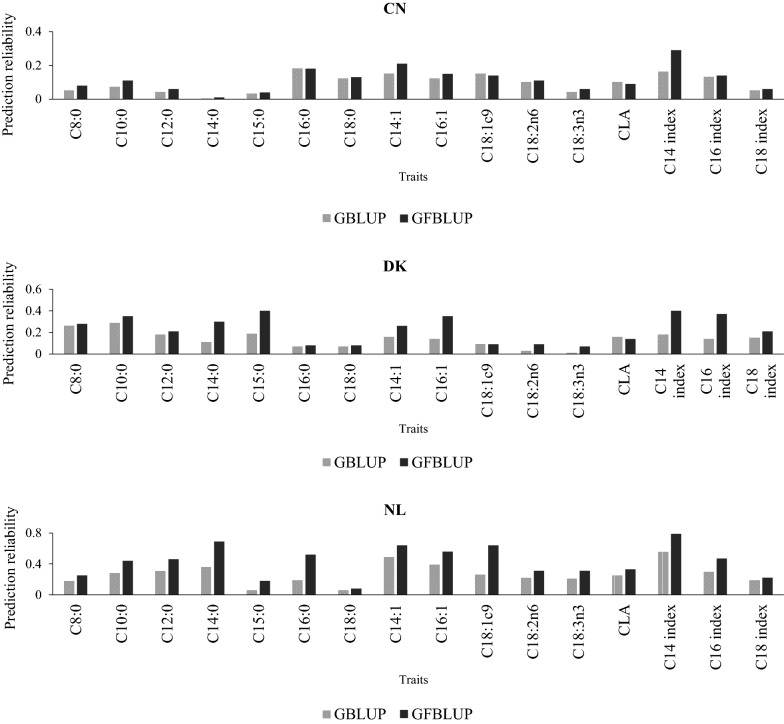
Genomic prediction accuracies using combined reference populations with traditional GBLUP and GFBLUP models in the Chinese, Danish and Dutch validation population

Substantial increases in prediction reliability were obtained using the GFBLUP model for multi-population reference sets compared to the combined GBLUP model (Fig. 2). Accordingly, an average gain in prediction reliability of 16% points was observed in the Dutch validation when the GFBLUP model was used compared to the traditional GBLUP with the combined reference population. Increases in prediction reliability were largest for C18:1c9 (∆ = 0.38), C16:0 (∆ = 0.33) and C14:0 (∆ = 0.33) in the validation for the Dutch Holstein. Similarly, average gains in reliability of 9% points in the Danish validation and 2.3% points in the Chinese validation populations were achieved by using the GFBLUP model compared to the GBLUP with the same reference population sizes. For the Danish validation, prediction reliabilities increased highly for C16 index (∆ = 0.23), C14 index (∆ = 0.22), C15:0 and C16:1 (∆ = 0.21). In the Chinese validation, the largest increase in prediction reliability obtained with the GFBLUP model was observed for C14 index (∆ = 0.13) followed by C14:1 (∆ = 0.06). Using the Dutch dataset as an example, we also studied the predictive ability of the GFBLUP model in a single-population reference setting (see Additional file 1), and the results showed an average increase of 12% points in prediction reliability with the GFBLUP model compared to the traditional GBLUP.

Whereas the increases in prediction reliability from the GFBLUP model varied between populations, sizable improvements for specific FA traits were consistent across the populations. For example, for the C14 index, gains in prediction reliability were 23% points in the Dutch, 22% points in the Danish and 13% points in the Chinese validation populations.

**Fig. 3:**
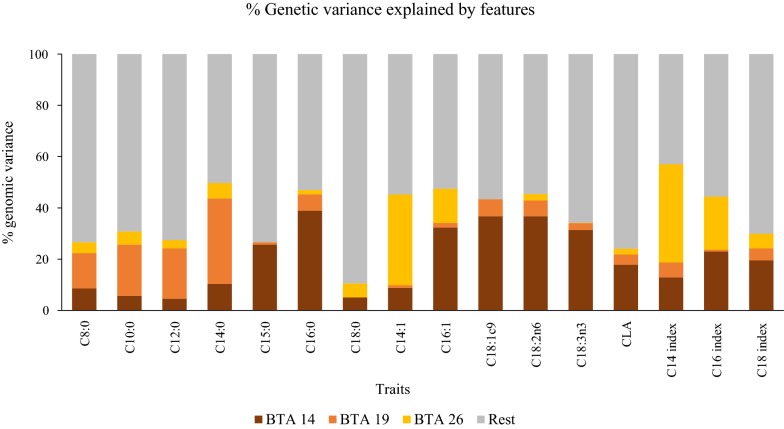
Proportion (%) of the genomic variance explained by genomic features fitted in the GFBLUP and the rest of genome-wide variants

In the combined-population analysis, the genomic features fitted in the GFBLUP model (BTA14, BTA19 and BTA26) collectively explained substantial proportions of the total genetic variance across the FA traits (Fig. 3). For instance for the C14 index, 57% of the genetic variance were explained by the genomic features, with BTA26 alone explaining 38.3% of the genetic variance. Similarly, sizable proportions of the total genetic variance for C14:0 (33.4%) and C10:0 (20%) were explained by BTA19 alone. Variants on BTA14 collectively explained 38.3 and 36.7% of the genetic variance for C16:0, and for C18:1n9 and C18:2n6, respectively. In contrast, for C18:0, BTA14 and 26 each explained 5% of the genetic variance.

**Fig. 4:**
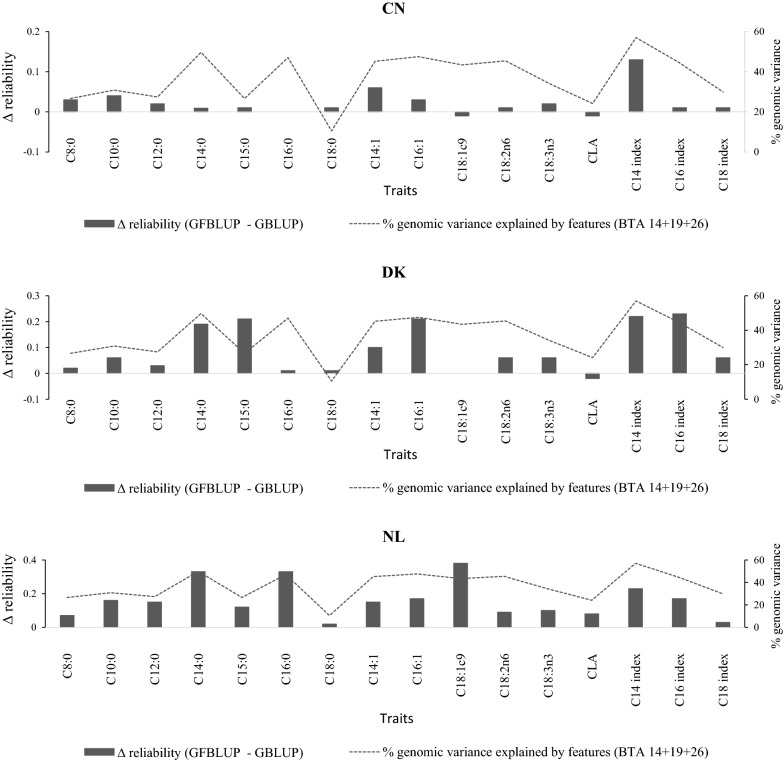
Relationship between the proportion of genetic variance explained by genomic features and gain in prediction reliability. Proportion of genetic variance explained by BTA14, 19 and 26 summed together (dotted lines, with values on the right of the y-axis) and change in prediction reliability using the GFBLUP model (on the left of the y-axis)

For most of the studied traits, the gain in prediction reliability obtained by using the GFBLUP model across the FA traits was related to the proportion of genomic variance explained by the fitted genomic features. Such trends were consistent across the validation populations especially for C18:0, CLA and C14 index. However, for C18:1c9 we observed no improvement in prediction reliability in the Danish population, and even a decline in the Chinese dataset, with the GFBLUP model in spite of the fact that a sizable proportion of the genetic variance was explained collectively by the fitted features (56.6%). The relationship between proportions of genetic variance explained by the genomic features and gains in prediction reliability from the GFBLUP model (vis-à-vis traditional GBLUP) is shown in Fig. 4. Scatterplots of the GBV that were predicted by using the GFBLUP model against the corrected phenotypes for all validation individuals from all replicates are in Additional files 2, 3 and 4 (see Additional files 2, 3, 4) for the validations in the Chinese, Danish and Dutch populations, respectively. The slope of the regression of corrected phenotypes on predicted GBV varied according to trait and population studied. Regression slopes were close to 1 for all traits except C14 (1.40) and C18:3n3 (1.42) in the Dutch validation across all scenarios. However, the values for most traits deviated from 1 in the Danish and Chinese populations and this deviation was especially high for C16:0, C18:1c9, C18:2n6 and C18:3n3 in the Danish and C14:0, C15:0 and C16 index in the Chinese population.

The prediction reliabilities reported here for each scenario are average values of five replicates. The differences in correlations between the corrected phenotypes and predicted GBV among the five replicates varied according to trait and population. The mean absolute deviation in the correlation values of the replicates from the reported average value ranged from 0.01 to 0.17 across traits in the different models for the Chinese validation, and from 0.03 to 0.10 in the Danish and from 0.04 to 0.21 in the Dutch validation.

## Discussion

### Combining reference populations

The population-specific genomic prediction for the FA traits studied, generally, resulted in low prediction reliabilities in the Chinese and Danish populations. For the Dutch validation, moderate prediction reliabilities were achieved for some of the FA traits. In general, the low prediction reliabilities with a population-specific reference reflect the well-established impact of a small reference population in genomic prediction reliability [37, 38]. However, prediction reliabilities were in general lower for the Chinese single-population prediction than for the Danish single-population prediction although the sizes of their reference populations were similar. This can be partially explained by the lower heritability estimates for the Chinese dataset for most of the FA traits than for the other datasets. The effect of a small reference population size is larger on low heritability traits for which a relatively large number of records will be required in the reference population to achieve high accuracies of GBV in unphenotyped animals [39]. Cows from a few sire-groups were used for the prediction in the Chinese and Dutch populations. Prediction accuracies obtained with such large half-sib groups might not be representative of those obtained with larger populations in which such a parental structure is less common. The distribution of haplotypes in a population comprising few sires will differ from that in a random population sample and the estimated SNP effects may be biased, to some extent, since they are conditioned on the haplotypes of the few sires represented in the reference population.

Combining reference populations resulted in a relatively sizable improvement in prediction reliability in the Danish and Dutch validation. Previous studies using either simulated [40] or real data [41] suggested that pooling data from different populations is beneficial for the accuracy of genomic predictions. However, the genetic distance between the populations/breeds that are combined [8], the marker density used, the genetic architecture of the traits [42], and the inconsistencies in allele substitution effects [43] might affect such advantages. A gain in prediction reliability of up to 9% points was obtained for some traits in the Chinese validation, whereas little or no increase was observed for some of the traits by adding the Dutch and Danish Holstein reference populations. This was unexpected given the genetic similarity and high consistency in genome-wide LD that exist between the populations combined [10, 11]. One possible reason for the limited benefits from combining the reference populations for the Chinese validation could be differences in SNP effects between the Chinese population, on the one hand, and the Dutch and Danish populations, on the other hand. Previously, in a joint GWAS using the same dataset, we (Gebreyesus et al. personal communication) found that effects of SNPs in the *DGAT1* and *SCD1* genes on BTA14 and 26, respectively, were smaller in the Chinese dataset than in the Dutch and Chinese datasets. Polymorphisms in *DGAT1* and *SCD1* underlie a substantial proportion of the genetic variation in most milk FA traits. Based on our previous GWAS results, we suggested that differences in feeding systems are the most likely source of significant differences in phenotypic means between the Chinese dataset, on the one hand, and the Dutch and Danish datasets, on the other hand. Fitting herd as a fixed effect accounts for differences due to management systems. However, such differences can introduce feed-by-genotype interactions that result in differences in the size of SNP effects. Inconsistencies in SNP effects have been shown to reduce the advantages from multi-population genomic prediction [43]. On the one hand, SNP effects estimated in a multi-population reference, which is dominated by Dutch Holstein cows (n = 1566) compared to the Danish (n = 614) and Chinese (n = 586) cows, are used to predict breeding values for the 100 Chinese validation animals. On the other hand, the corrected phenotypes from the single-population analysis for these validation animals reflect the SNP effects in the Chinese population. With such differences in the estimates of SNP effects for the Chinese population compared to the others, the correlations between breeding values estimated by using the multi-population reference sets and the corrected phenotypes, i.e., prediction accuracies, are expected to be low. This also leads us to expect smaller contributions from the Chinese reference population for the gains in prediction reliability observed for the Dutch and Danish validations using the combined reference predictions.

### Incorporating GWAS results in prediction models

In this study, we also implemented a GFBLUP model that considered BTA14, 19 and 26 as genomic features and allowed separate random genetic effect components for these regions. In general, implementation of this GFBLUP model resulted in further improvements in prediction accuracy for most of the traits across the validation populations. However, the level of improvement varied across the populations with the smallest improvement observed for the Chinese validation.

The relative gain in prediction reliability with the GFBLUP model across the FA traits appears to be related to the proportion of genetic variance that is collectively explained by the features considered. For instance, the gain in prediction reliability from the GFBLUP model was highest for the C14 index, in all three populations, for which more than half of the genomic variance was explained by the genomic features collectively, i.e., BTA14, 19 and 26. Likewise, smaller increases in prediction reliability across the populations were computed for C18:0, which had the smallest proportion of variance explained by BTA14, 19 and 26. However, this pattern is mainly limited to the Danish and Dutch validation since the gains in prediction reliability from the GFBLUP were generally low in the Chinese validation regardless of the proportion of variance explained by the features.

Different methods have been suggested to incorporate biological information in genomic prediction models. For instance, MacLeod et al. [13] introduced the Bayes RC model, an extension of the Bayes R model [44], to incorporate biological information by defining classes of SNPs that are likely to be enriched for causal variants. Similarly, Brøndum et al. [45] presented Bayesian prediction models based on genome-position specific priors, whereas Gebreyesus et al. [46] introduced hierarchical Bayesian models based on the clustering of adjacent SNPs to exploit heterogeneous (co)variance patterns. However, with few exceptions (e.g. Zhang et al. [47]), most of these models are implemented within a Bayesian framework that is computationally demanding. Hence, their applicability in routine evaluations is limited. GBLUP is straightforward to implement and estimated GBV are similar to those estimated in the BLUP approach [6] since the method, i.e., assumption of a normal distribution of QTL effects, is equivalent to the BLUP approach used in traditional breeding programs [28]. Thus, its simplicity and lower computational burden have made GBLUP a method of choice in routine genetic evaluations. Therefore, implementing biological information-augmented approaches in GBLUP models are closer to practical implementation in the breeding industry.

Our findings show that improvement in prediction reliability from multi-population prediction using the GFBLUP model was substantial and consistent across populations for some of the traits analyzed. These include the C14 index and, to some extent, C14:1 and C16:1 FA. Saturated FA in milk, in particular C12:0, C14:0 and C16:0 are frequently associated with increases in serum cholesterol in humans, and this has been the basis for the development of the atherogenicity index: (C12 + 4·C14 + C16)/(MUFA + n3 PUFA + n6 PUFA) [48]. We have shown that by pooling data and incorporating biological information, it is possible to predict genetic merits for the composition of such FA with accuracies as high as 0.79 in spite of the limited size of the reference populations used. These findings highlight the possibility of implementing selective breeding to modify the bovine milk FA composition, although large-scale phenotyping for these traits is still a challenge. Our findings also indicate that genomic prediction for small-scale recorded traits might benefit from international collaborations for access to data across populations.

## Conclusions

In this study, we compared the accuracies of genomic prediction for the detailed milk fat composition traits using a multi-population reference and a model incorporating GWAS findings with those obtained with traditional GBLUP models in a single population scenario. Our results indicate that pooling multi-population data and implementing prediction models augmented with biological information can enable prediction of genetic merits for the small-scale recorded bovine milk FA composition traits with reasonable accuracies. High prediction reliabilities were estimated for some of the FA traits using the multi-population reference and a GFBLUP model, e.g. 0.79 for the C14 index, 0.69 for C14:0 and 0.64 for C18:1c9, which indicates that the modification of the milk fatty acid composition through selective breeding could be considered in spite of the current limitations in large-scale phenotyping.

## Additional files

**Additional file 1.** Genomic prediction reliability in the Dutch population using single- and combined-population GBLUP and GFBLUP. Bar plots of genomic prediction reliability (y-axis) for all the FA traits (x-axis) in the Dutch population with single-population GBLUP, single-population GFBLUP and combined reference population GFBLUP models.

**Additional file 2.** Plots of predicted GBV against corrected phenotypes from the GFBLUP model in the Chinese validation. Scatterplots of predicted GBV (y-axis) against corrected phenotypes (x-axis) from the GFBLUP model in the Chinese population using all five validation sets.

**Additional file 3.** Plots of predicted GBV against corrected phenotypes from the GFBLUP model in the Danish validation. Scatterplots of predicted GBV (y-axis) against corrected phenotypes (x-axis) from the GFBLUP model in the Danish population using all five validation sets.

**Additional file 4.** Plots of predicted GBV against corrected phenotypes from the GFBLUP model in the Dutch validation. Scatterplots of predicted GBV (y-axis) against corrected phenotypes (x-axis) from the GFBLUP model in the Dutch population using all five validation sets.
