# Supplementary figures and images for: Reliability of genomic prediction for milk fatty acid composition by using a multi-population reference and incorporating GWAS results
Source: Genet Sel Evol. 2019 Apr 27;51:16. doi: 10.1186/s12711-019-0460-z (PMC6487064; doi:10.1186/s12711-019-0460-z)

Prediction reliabilities in Dutch validation

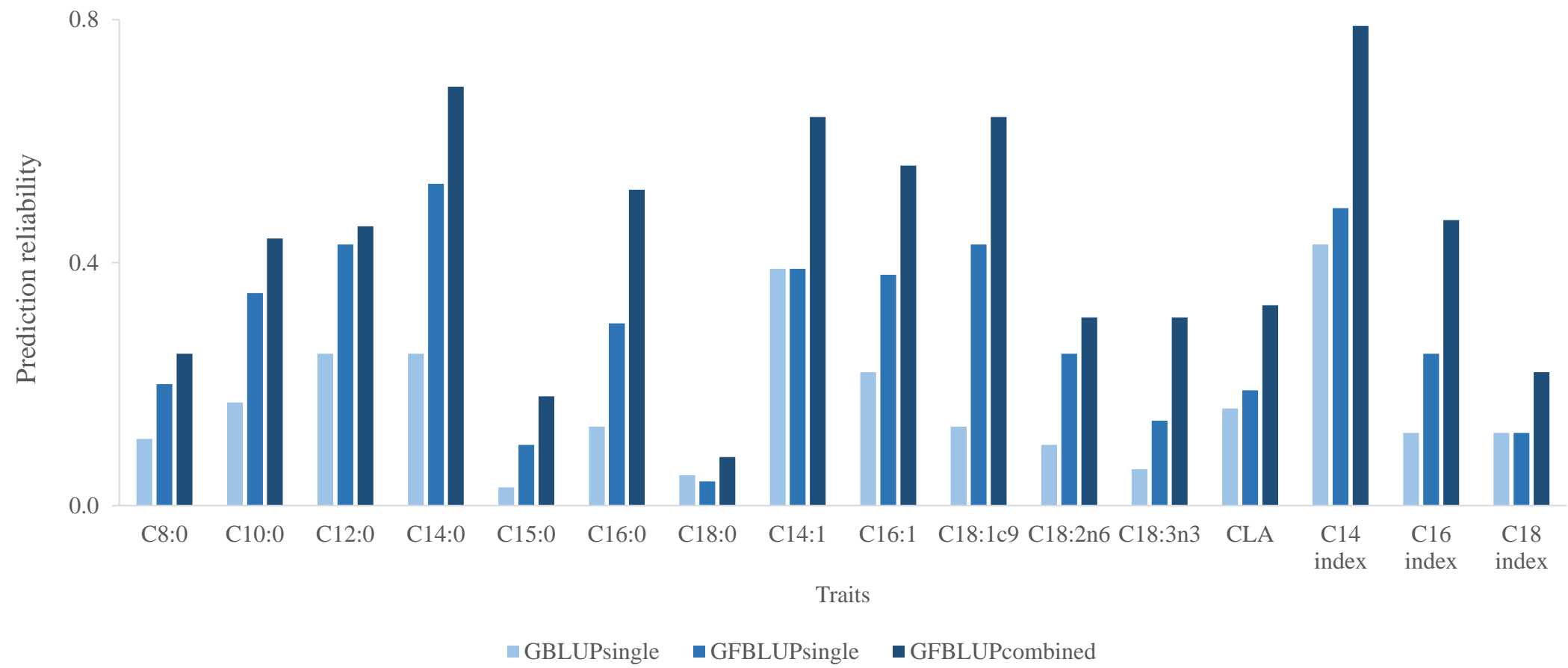

Supplement: Supplementary file 1 — Additional file 1. Genomic prediction reliability in the Dutch population using single- and combined-population GBLUP and GFBLUP. Bar plots of genomic prediction reliability (y-axis) for all the FA traits (x-axis) in the Dutch population with single-population GBLUP, single-population GFBLUP and combined reference population GFBLUP models. [file 12711_2019_460_MOESM1_ESM.pdf]

C8:0

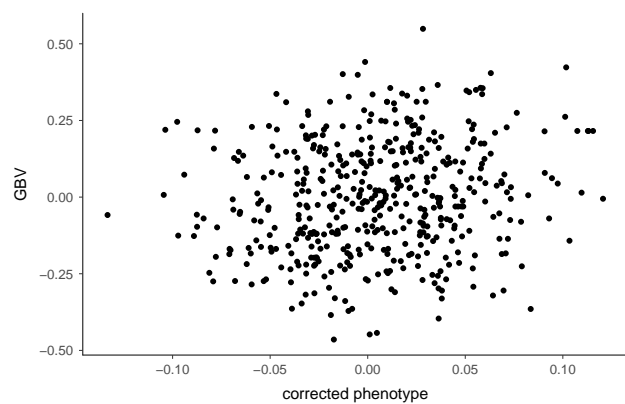

C10:0

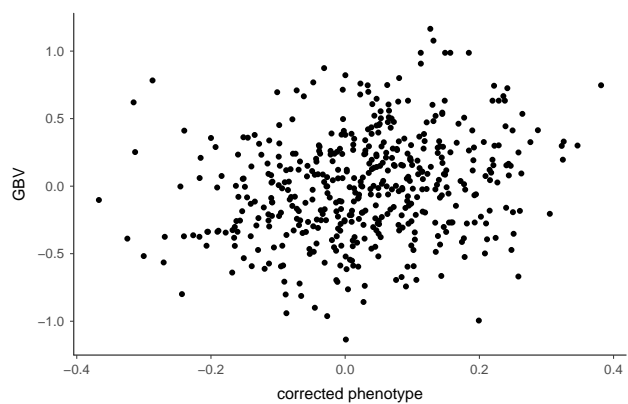

C12:0

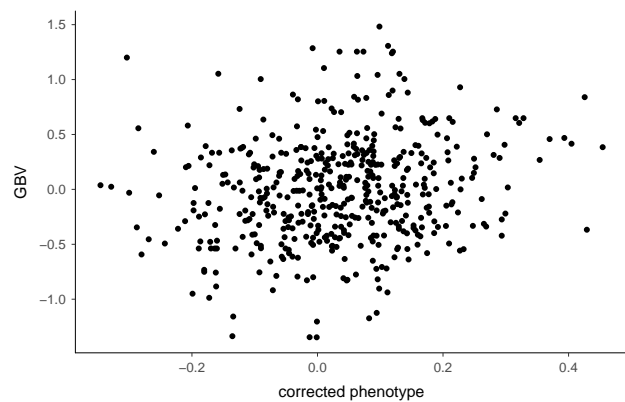

C14:0

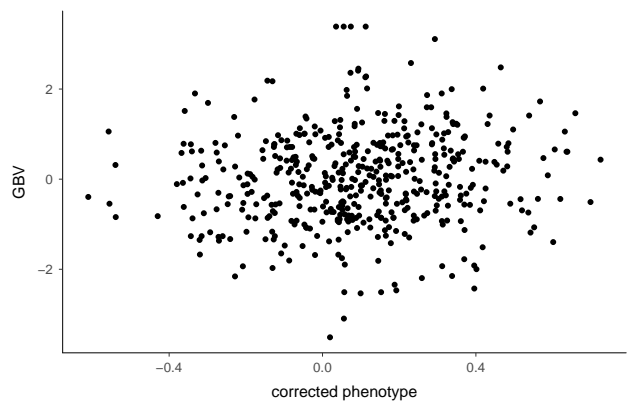

C15:0

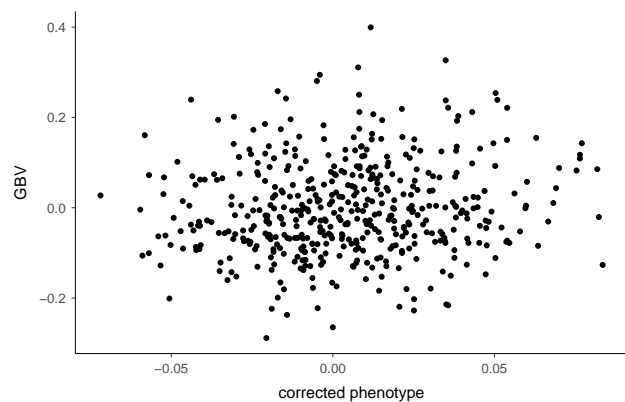

C16:0

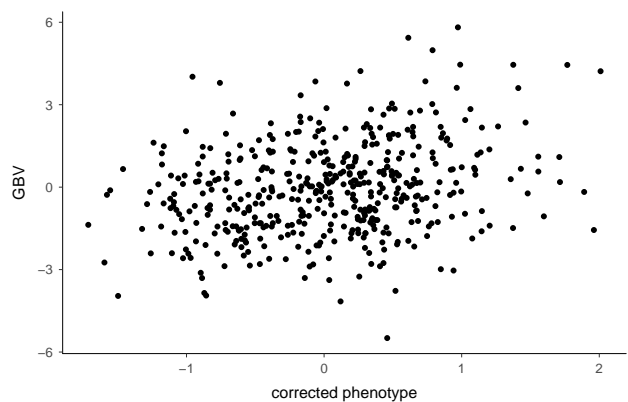

C18:0

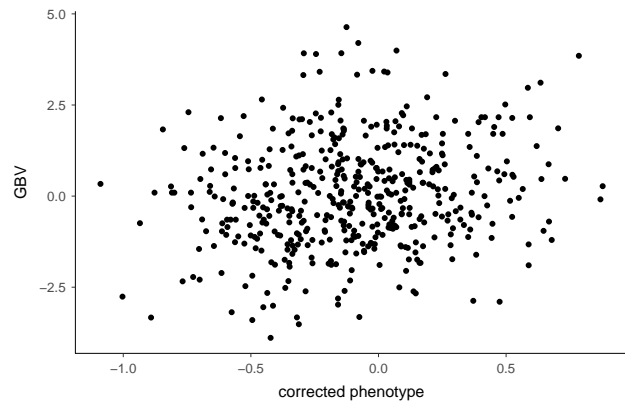

C14:1

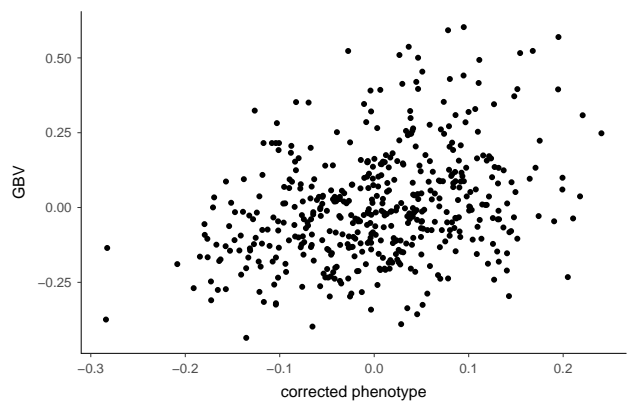

C16:1

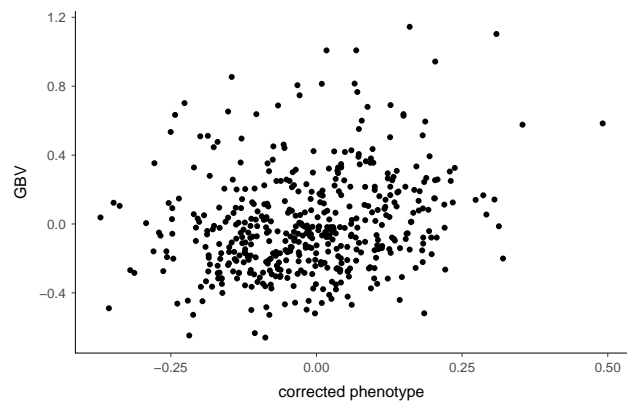

C18:1c9

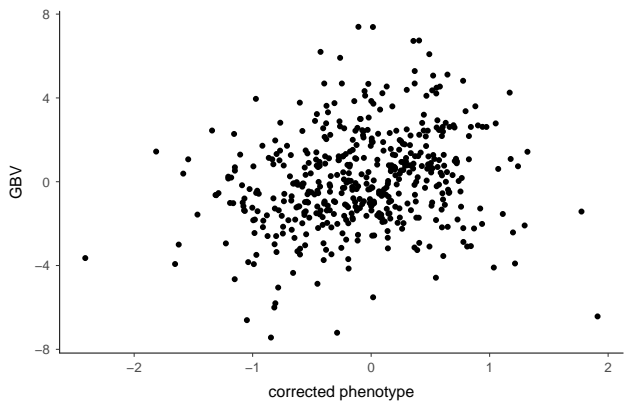

C18:2n6

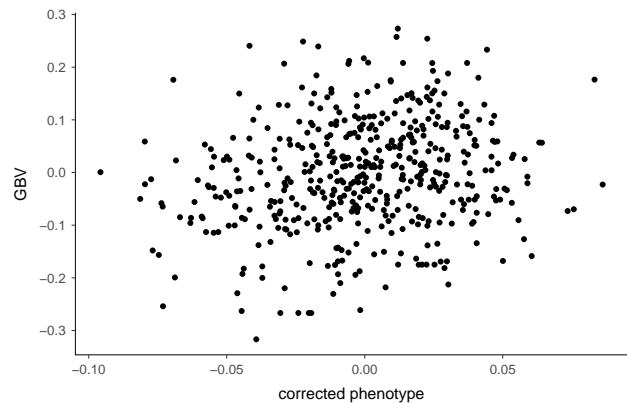

C18:3n3

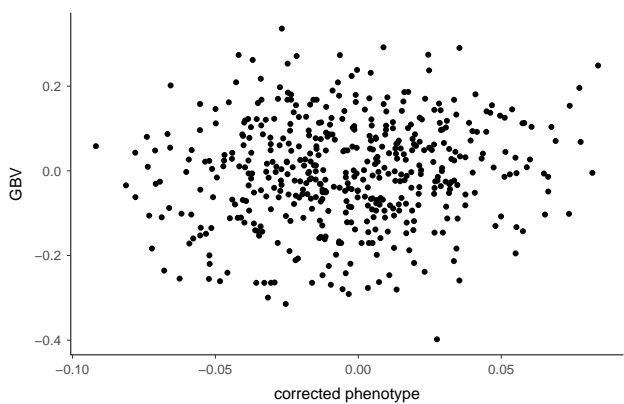

CLA911

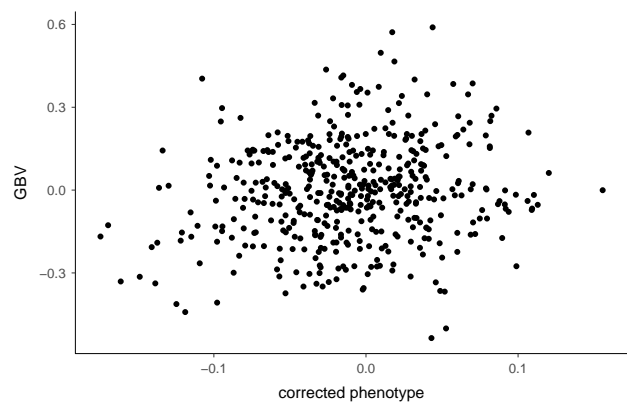

C14Index

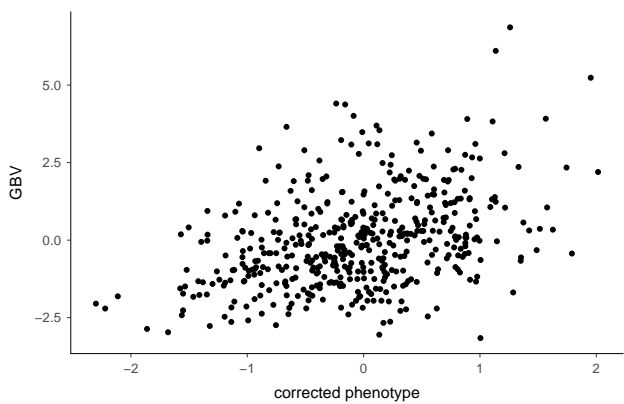

C16Index

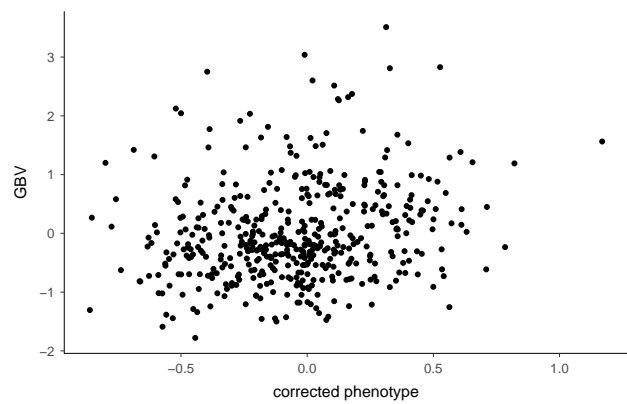

C18Index

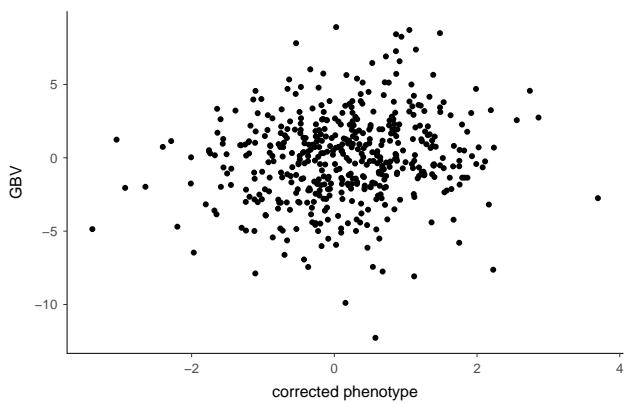

Supplement: Supplementary file 2 — Additional file 2. Plots of predicted GBV against corrected phenotypes from the GFBLUP model in the Chinese validation. Scatterplots of predicted GBV (y-axis) against corrected phenotypes (x-axis) from the GFBLUP model in the Chinese population using all five validation sets. [file 12711_2019_460_MOESM2_ESM.pdf]

C8:0

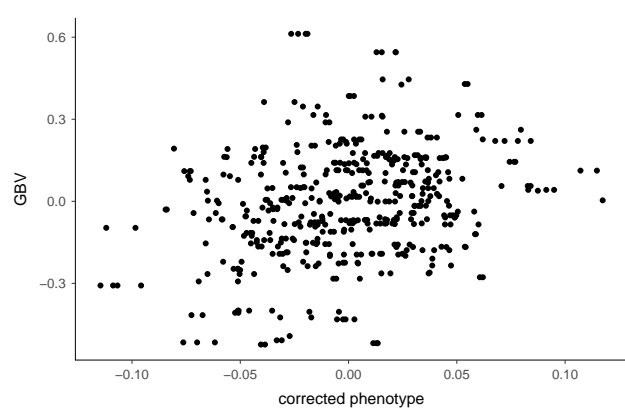

C10:0

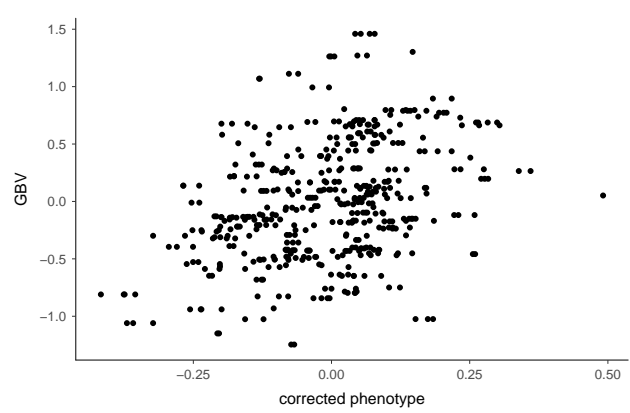

C12:0

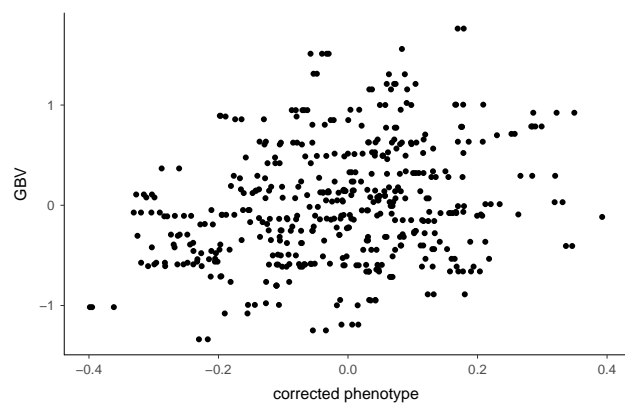

C14:0

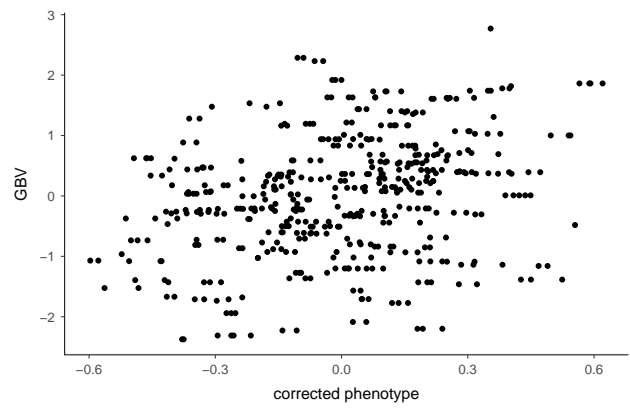

C15:0

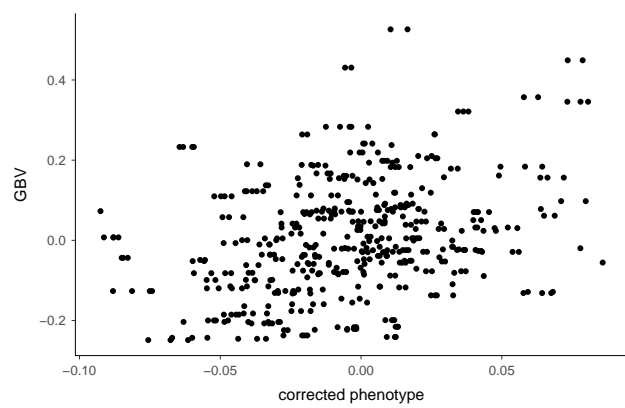

C16:0

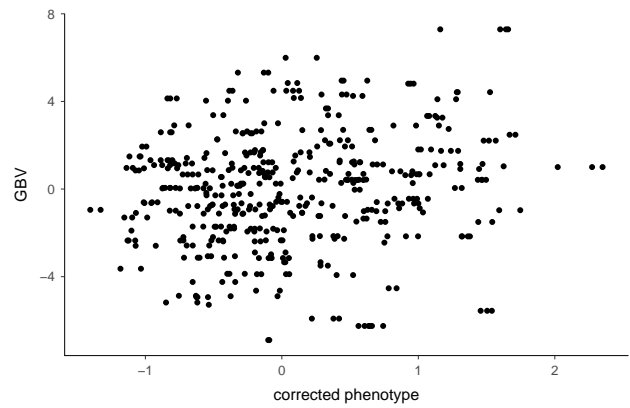

C18:0

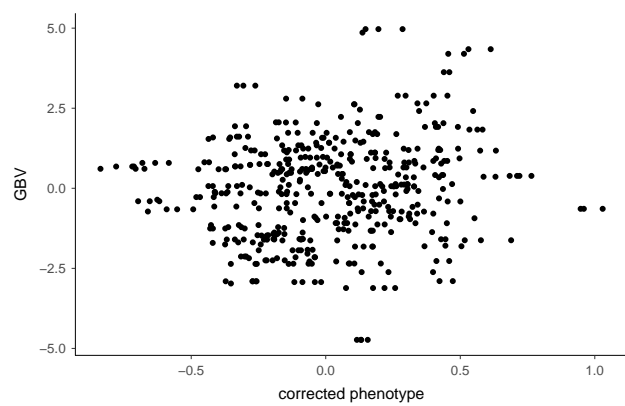

C14:1

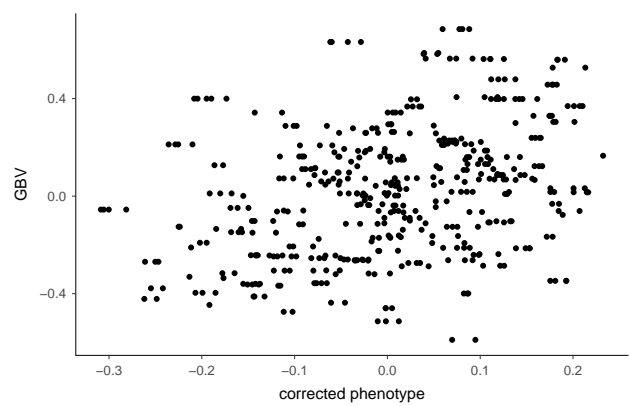

C16:1

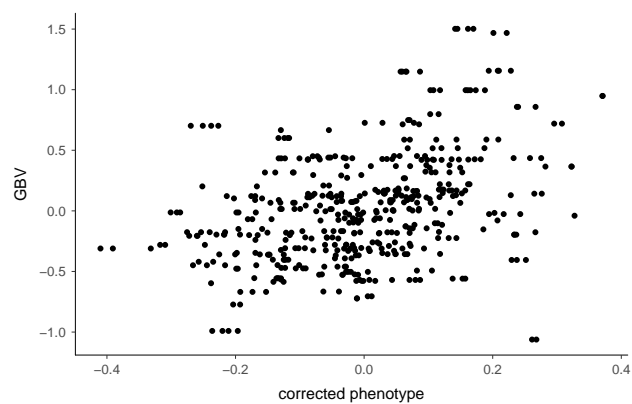

C18:1c9

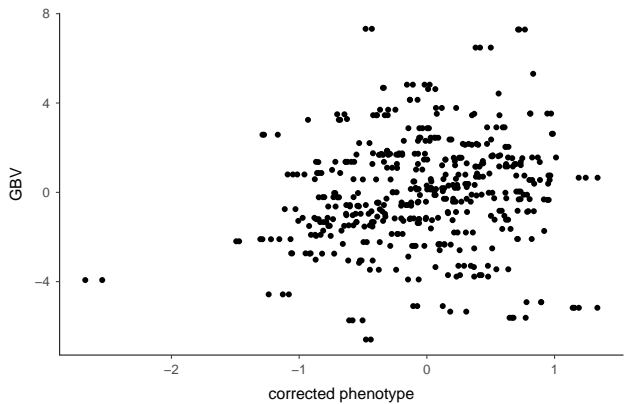

C18:2n6

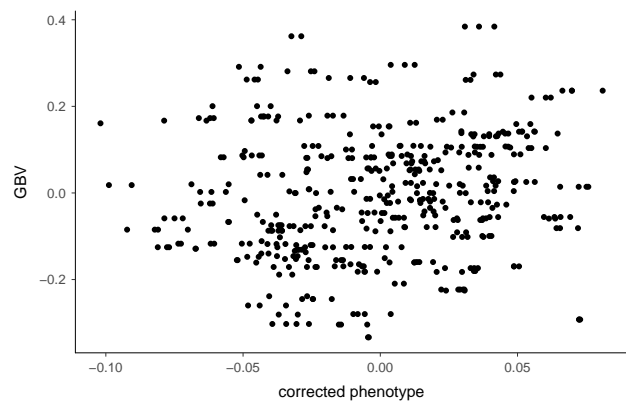

C18:3n3

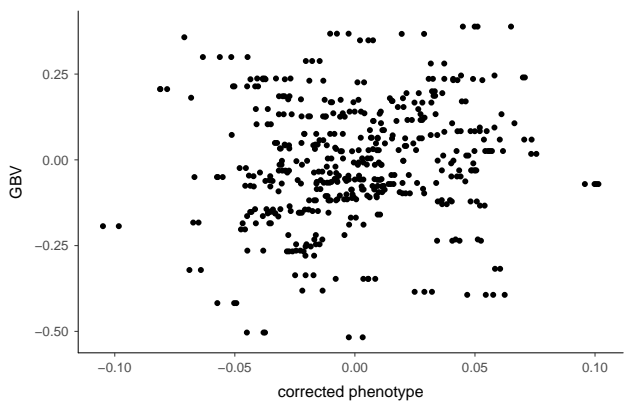

CLA911

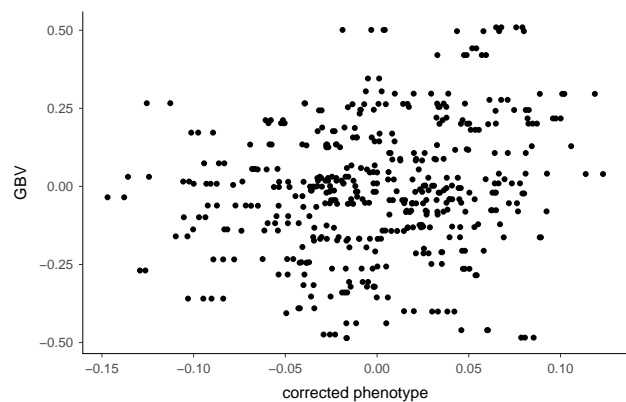

C14Index

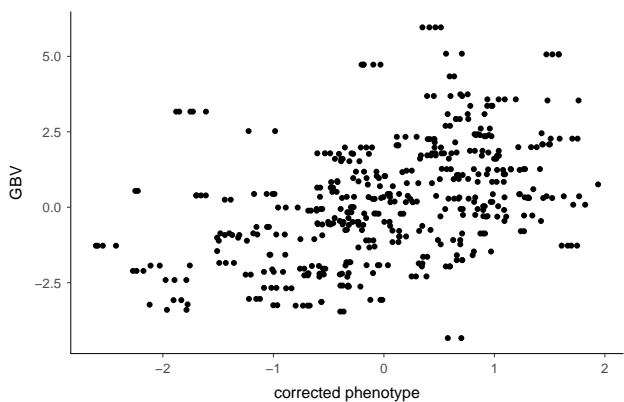

C16Index

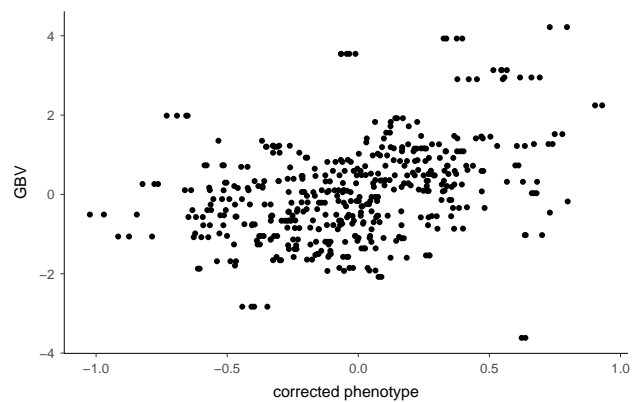

C18Index

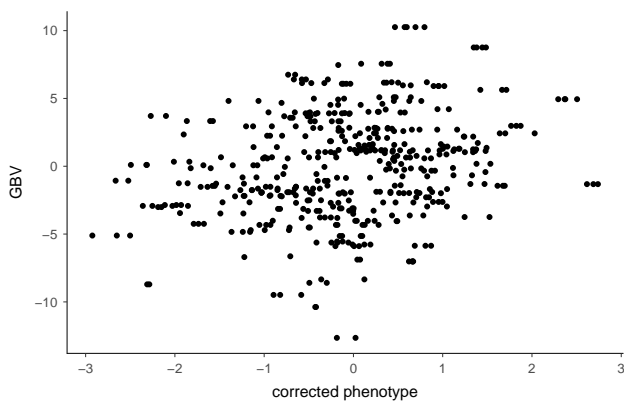

Supplement: Supplementary file 3 — Additional file 3. Plots of predicted GBV against corrected phenotypes from the GFBLUP model in the Danish validation. Scatterplots of predicted GBV (y-axis) against corrected phenotypes (x-axis) from the GFBLUP model in the Danish population using all five validation sets. [file 12711_2019_460_MOESM3_ESM.pdf]

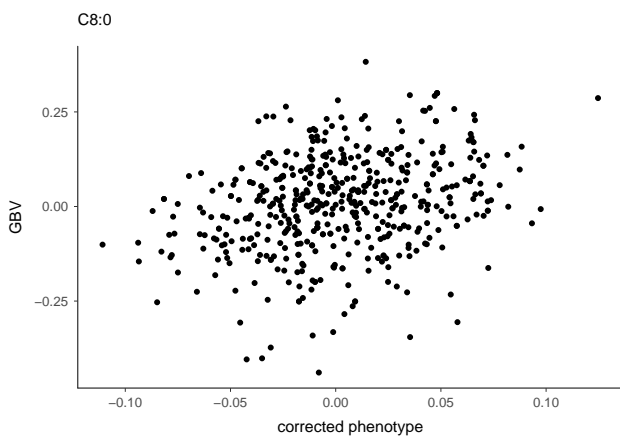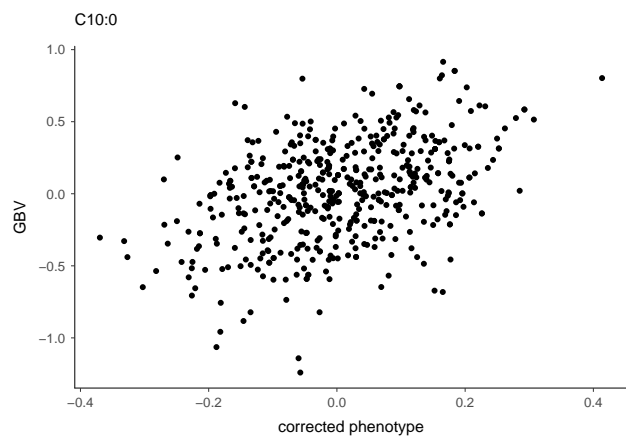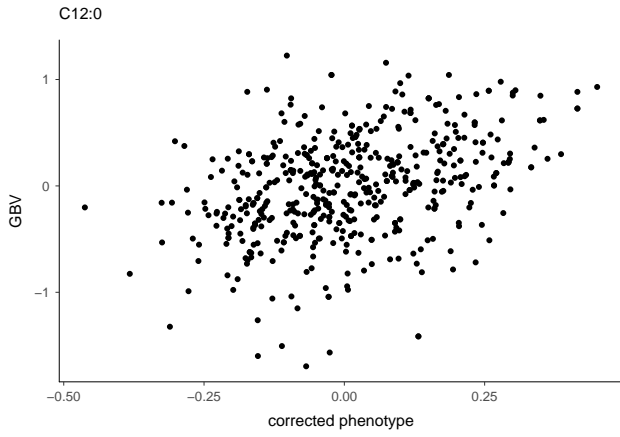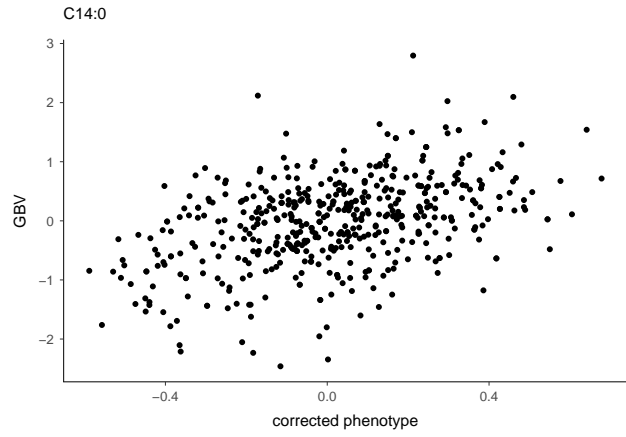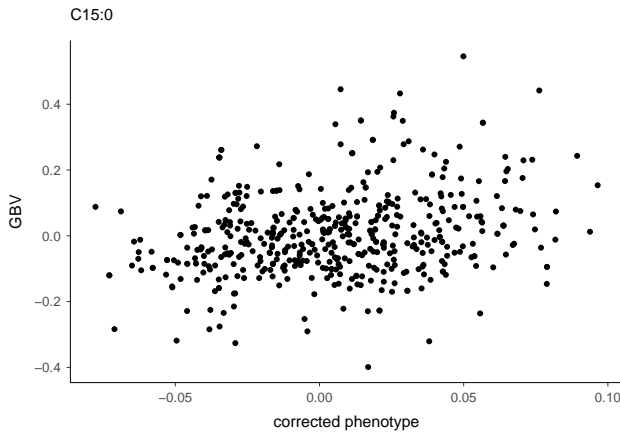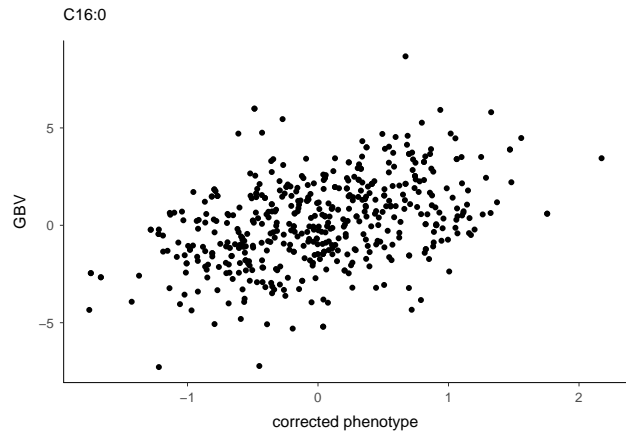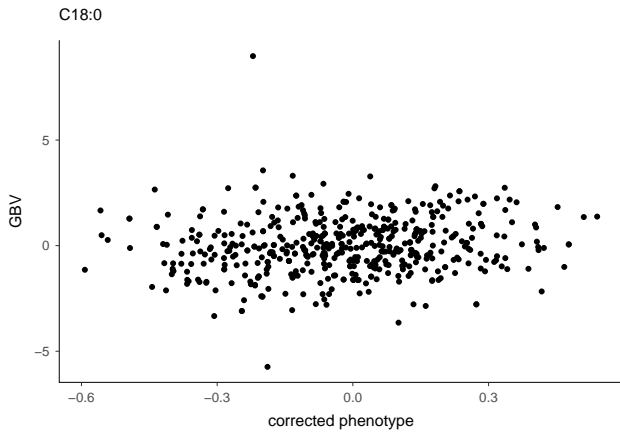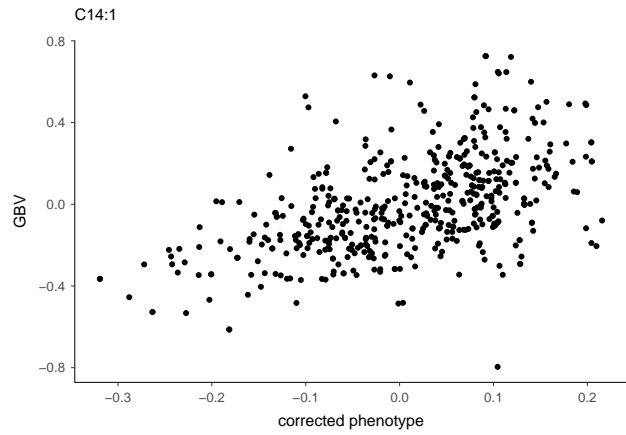

C16:1

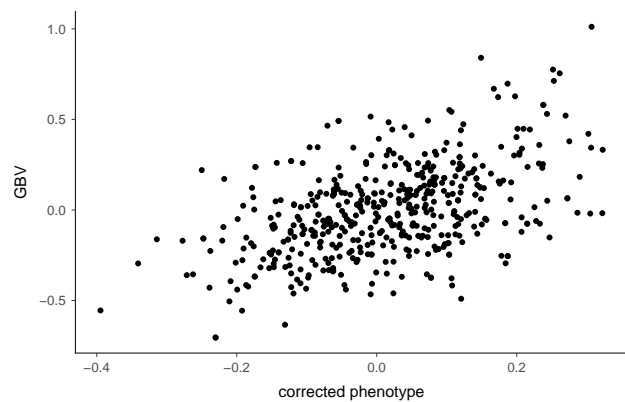

C18:1c9

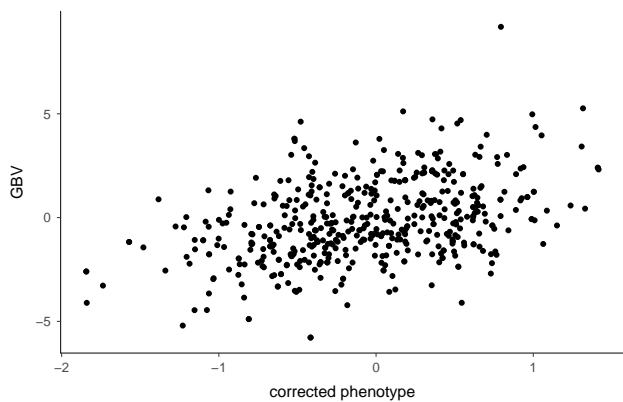

C18:2n6

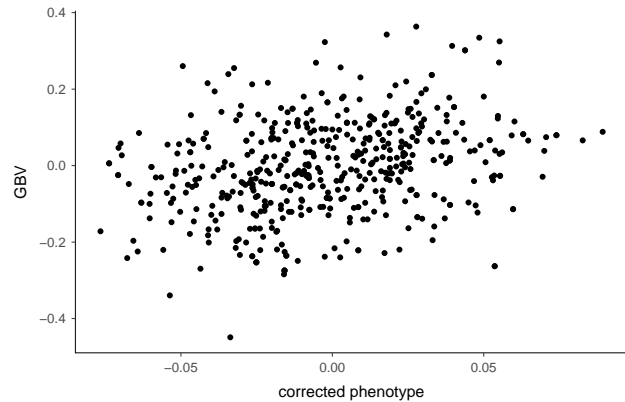

C18:3n3

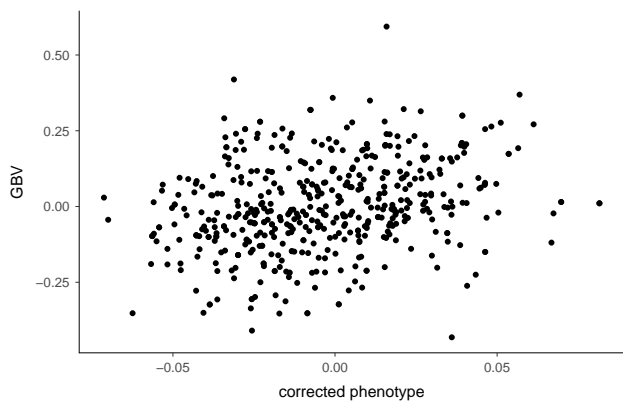

CLA911

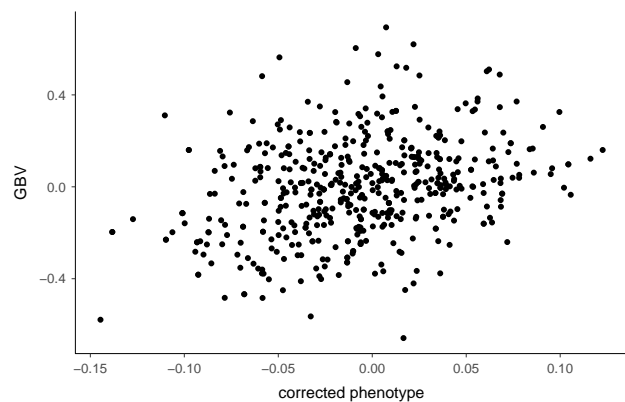

C14Index

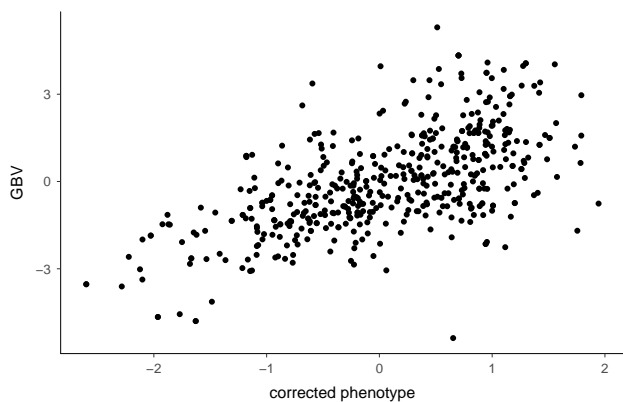

C16Index

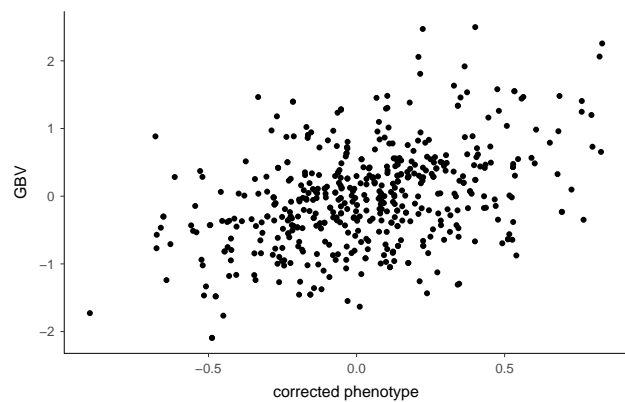

C18Index

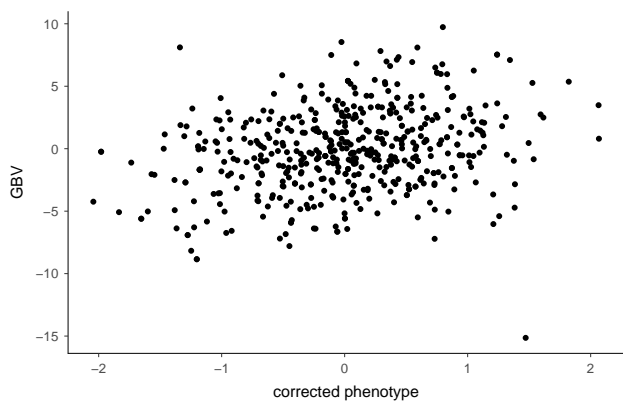

Supplement: Supplementary file 4 — Additional file 4. Plots of predicted GBV against corrected phenotypes from the GFBLUP model in the Dutch validation. Scatterplots of predicted GBV (y-axis) against corrected phenotypes (x-axis) from the GFBLUP model in the Dutch population using all five validation sets. [file 12711_2019_460_MOESM4_ESM.pdf]
